# Multi-walled carbon nanotube-induced inflammatory response and oxidative stress in a dynamic cell growth environment

**DOI:** 10.1186/1754-1611-6-22

**Published:** 2012-11-13

**Authors:** Hemang Patel, Soonjo Kwon

**Affiliations:** 1 Department of Biological Engineering, Utah State University, 4105 Old Main Hill, 84322-4105, Logan, UT, USA

**Keywords:** MWCNTs, Dynamic cell culture, IL-8, ROS, GSH

## Abstract

**Background:**

Rapid increase in multi-walled carbon nanotube (MWCNT) production for their industrial and biomedical applications has led to concerns over the effects of MWCNTs on human health and the environment. Both animal and *in vitro* studies have provided important findings about MWCNT-induced effects on the lung cells or tissues. *In vitro* studies have provided a considerable amount of fundamental information on MWCNT-induced effects on the specific lung cells. However, the cell culture systems used in those studies were limited by the absence of dynamic nature of lung tissues. We hypothesized that MWCNT-induced cellular responses such as proliferation, inflammation, and oxidative stress under dynamic cell growth environment may differ from those under static cell growth environment.

**Results:**

In this study, we used a dynamic cell growth condition to mimic mechanically dynamic environment of the lung and characterized interleukin 8 (IL-8), reactive oxygen species (ROS), glutathione (GSH), and cell proliferation for three days following exposure of MWCNTs at different concentrations (5, 10, and 20 μg/ml) to A549 cell monolayer under both static and dynamic cell growth conditions. Our results demonstrated the distinct differences in the levels of inflammatory response and oxidative stress between static and dynamic cell growth conditions.

**Conclusions:**

In conclusion, the dynamic cell growth system used in this study provided important changes in cellular responses that were not found in the static cell growth system and were similar to animal studies. The dynamic cell growth system can be considered as a viable alternative to *in vivo* test system in combination with existing *in vitro* static cell growth systems to evaluate the effect of MWCNTs on cellular responses in the respiratory system.

## Introduction

Rapid advancement in the field of nanotechnology has given birth to various types of nanomaterials with unique mechanical, thermal, and electrical properties. Carbon nanotubes (CNTs) have shown tremendous potentials for their use in diverse applications due to their unique electrical and mechanical properties. Due to their promising potentials in industrial and medical applications, the demands for CNT production has steadily increased in last few years and expected to dramatically increase in near future. It was reported that the global market for CNT production in 2009 was $103 million, which has been projected to reach $1 billion by 2014 with compound annual growth rate of 58.9% [1]. MWCNTs consist of many hollow cylinders of carbon atoms inside one another, which enhance the mechanical, thermal and electronic properties through the increase in higher carbon atoms integration and bigger surface area. Along with the increasing demand in MWCNT production, natural eco-system contamination and human exposure through occupational and medical applications have been expected due to the nano-scale size and non-degradability of MWCNTs [2-4]. In recent animal studies, MWCNT’s high penetrative nature, long retention time, and capability of initiating pathological response inside the lung have been serious concerns [5-10]. Many studies have emphasized on understanding MWCNT-induced effects on either respiratory or dermal systems. The recent animal studies have highlighted the higher retention of MWCNTs in alveoli region after 6 month exposure and the probability of penetrating to nearby tissues [2,5,10]. Most of the animal studies focused on investigating long term effects of MWCNT exposure, and showed highly penetrating nature of MWCNTs and increased macrophage assisted clearance in conjunction with elevated inflammation levels [5,7,8,11,12]. Other studies demonstrated the increased levels of cytotoxic and inflammatory response even within a day following exposure of MWCNTs [5,6,8,10,13,14]. The systemic approach for the evaluation using animals was helpful to characterize the whole lung response but had a limitation in investigating MWCNT interaction with each individual cell type in the lung. The *in vitro* cell culture models provided the fundamental information regarding MWCNT-induced effects on individual cell types in the lung. Lung epithelial cells act as a barrier at the interface between surrounding air and lung tissues in respond to exogenous particles such as air-pollutants including CNTs. MWCNTs induce a variety of effects including increased inflammatory response, DNA damage, and cellular apoptosis in A549 cells (immortal human alveolar epithelial cell line), normal human bronchial epithelial cells and rat lung epithelial cells [15-18]. These studies using specific cell type have provided abundant on the response of epithelial cells to external perturbations, but these systems are limited by the absence of dynamic nature of lung tissues [19]. The lung exists in a mechanically active environment, where different amounts of circumferential and longitudinal expansion and contraction occurred during breathing movements. Patel and co-workers recently showed the differences in cellular responses to air pollutants between dynamic and static cell growth environments, and demonstrated that implementing dynamic cell growth conditions was more close approximation of *in vivo* conditions. [20]. Such changes might have resulted from the altered interactions between cells and air pollutants under mechanically active cell growth environment. In this study, we evaluated the effect of MWCNT exposure on cellular responses under both static and dynamic cell growth environments at different concentrations (5, 10 and 20 μg/ml) of MWCNTs. We hypothesized that MWCNT exposure under dynamic cell growth environment of cells may alter its interaction with cells, and affect the levels of cell proliferation (total cell protein), cellular inflammation (IL-8), and oxidative stresses (ROS and GSH). To test our hypothesis, we used Flexcell Tension Plus 4000T system (Flexcell International, PA) for simulating dynamic cell growth environment, similar to normal breathing condition (5% of equibiaxial surface elongation at the frequency of 0.2 Hz) in the lung [21,22]. The dynamic *in vitro* culture system of A549 cells was used to investigate MWCNT-induced effects on cell proliferation, IL-8, ROS, and GSH. This study will provide one of the alternative ways to evaluate nanoparticle-induced effects on human respiratory systems and a detailed insight for the development of a viable alternative to existing static *in vitro* or *in vivo* tests.

## Materials and methods

### Cell culture

**Figure 1:**
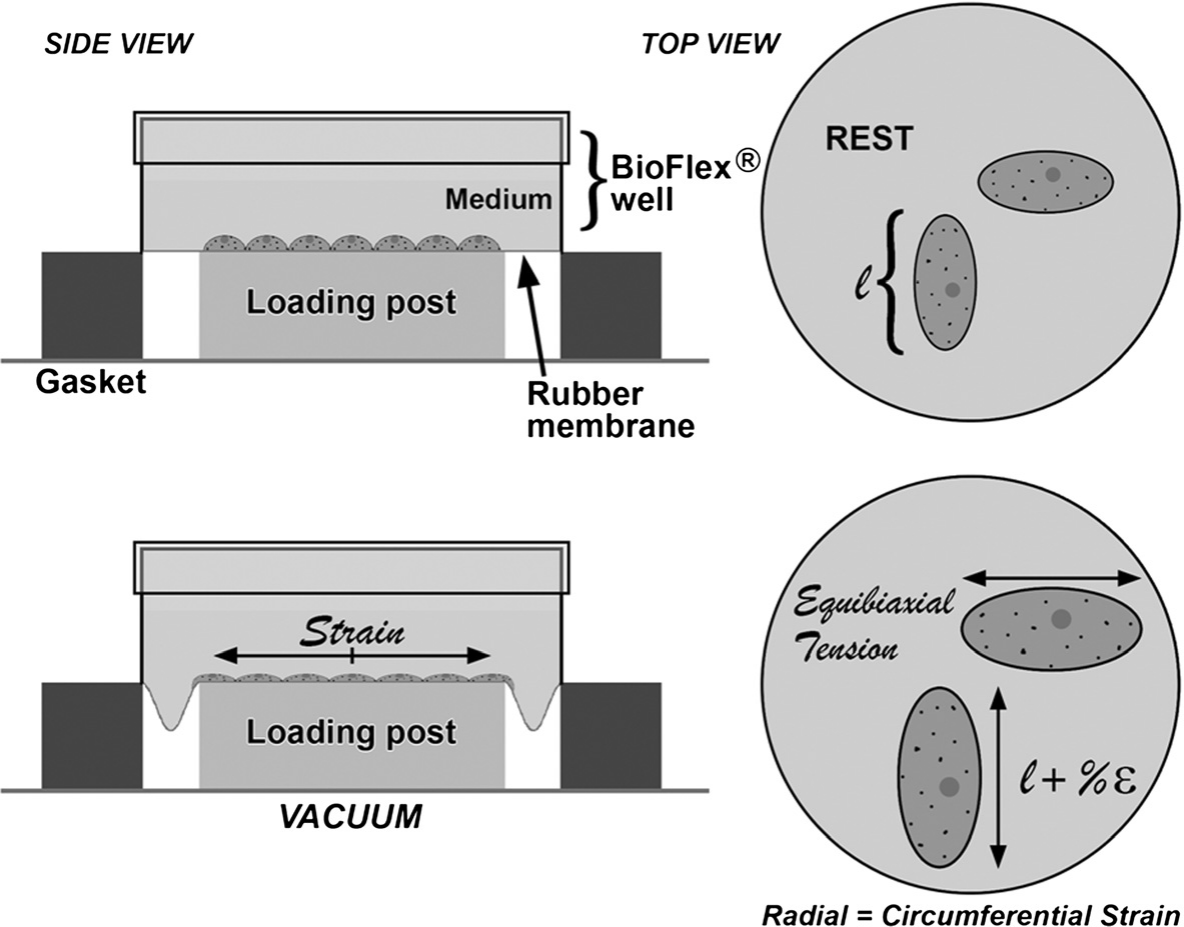
**Schematic Diagram of Flexcell Tension PlusTM 4000T system.** A computer driven system controls the pressure of vacuum, which pulls down the silastic membrane around loading post and implements the circumferential strain in cell culture growing on top of the loading post. During the course of dynamic cell growth, cyclic stretching was applied to silastic well bottoms of BioFlex plates to attain 5% surface elongation at the frequency of 0.2Hz, which corresponds to 45% of the total lung capacity similar to normal breathing condition in the lung.

Type II alveolar basal epithelial cells of human origin (A549) were purchased from ATCC (Manassas, VA). A549 is epithelial-like in morphology and originates from a human lung carcinoma patient. The cells were seeded at 3×10^5^ cells/well onto six well BioFlex plates (Flexcell International, PA) containing 2 ml of F-12k culture medium, which was supplemented with 1% penicillin streptomycin (Invitrogen, CA) and 10% fetal bovine serum (Thermo Fisher Scientific, UT). After cells reached confluence, in 48 hours of seeding, they were exposed to MWCNTs at 5, 10, and 20μg/ml and then grown in either static or dynamic (cyclic equibiaxial deformation) condition. The dynamic cell growth condition was implemented using Flexcell Tension Plus 4000T system, which used vacuum pressure to apply cyclic strain to cells cultured on BioFlex plates. Schematic diagram of Flexcell Tension PlusTM 4000T system was included in Figure 1. During the course of dynamic cell growth, cyclic stretching was applied to silastic well bottoms of BioFlex plates to attain 5% surface elongation at the frequency of 0.2Hz, which corresponds to 45% of the total lung capacity similar to normal breathing condition in the lung [21].

### MWCNT solution preparation

MWCNTs (length: 0.5-2 μm, outer diameter: 20–30 nm, inner diameter: 5–10 nm, and purity: >95 weight percentage (wt %)) were obtained from Cheap Tubes Inc., VT (SKU # 030404). MWCNT powder used in this study contained about 5 wt % of impurities including carbon black (3.34 wt %), iron (0.24 wt %), nickel (0.94 wt %), and chlorine (0.47 wt %). The stock solution for MWCNTs was prepared by suspending 50.5 mg of MWCNTs in 30 ml of sterile deionized water with 10.08 mg of polyvinylpyrrolidone (PVP). To breakdown the agglomerates and achieve better suspension of MWCNTs, stock solution was sonicated at 60 watts for 30 minutes with a 30 second cooling time per minute on ice using a Sonicator 3000 (MIsonix, Farmingdale, NY). To achieve exposure concentrations of 5, 10, and 20 μg/ml, appropriate amount of stock solution was added to each cell culture media. Right before the exposure study, MWCNT-containing cell culture media were sonicated for 5 minutes with a 30 second cooling time per minute on ice to make uniform suspension.

### MWCNT exposure

F-12k media containing different concentrations (5, 10, and 20 μg/ml) of MWCNTs were added to A549 monolayers when cells were confluent. Immediately after adding MWCNT-containing cell culture media, cells were grown in either static or dynamic condition for different exposure time (24, 48, and 72 hours). The dynamic cell growth condition was simulated by growing cell monolayers under continuous cyclic equibiaxial deformation with 5% surface area change at 0.2 Hz, which was similar to normal breathing *in vivo*[21]. Following each exposure time, media and cell lysate samples were collected and immediately stored in aliquots at −80°C until they were analyzed. All samples were analyzed immediately once thawed. Media supernatant samples were used to measure the level of IL-8. Cell lysate samples were used to measure the level of total protein, ROS, and GSH.

### IL-8 measurement

IL-8 was measured from media supernatant of A549 cell culture using ELISA prepared with IL-8 human antibody pair and buffer kit (Invitrogen, CA). The unit of IL-8 measurement was picograms/milliliter (pg/ml).

### Reactive oxygen species (ROS) measurement

ROS level was measured from the cell lysate of A549 cells using de-acetylated probe 2^′^,7^′^-dichlorofluorescin (H_2_DCF) based fluorescence assay to characterize the cellular level of oxidative stress. The H_2_DCF was prepared from 2^′^,7^′^-dichlorodihydrofluorescein diacetate (H_2_DCF-DA) by alkaline hydrolysis using NaOH [23]. 500 μl of 1 mM H_2_DCF-DA was added to 2 ml of 0.01N NaOH and hydrolyzed into H_2_DCF at room temperature for 30 min. The prepared H_2_DCF solution was neutralized by adding 10 ml of 25 mM NaH_2_PO_4_ and adjusting the pH of the solution to 7.4. Right after pH adjustment, 40 μM H_2_DCF solution was kept on ice or at 4°C until used. Fresh H_2_DCF solution was prepared before each ROS measurement to avoid molecular probe deterioration. To perform the ROS measurement, 20 μl of cell lysate was incubated with 50 μl of 40 μM H_2_DCF and 130 μl of 40 mM Tris–HCl, pH 7.4 for 10 min at 37°C, which initiated ROS facilitated H_2_DCF oxidization to 2′,7′-dichlorofluorescein (DCF). Level of DCF was measured using Synergy 4 series multiwell-plate fluorometer (Biotek, VT), which was set at an excitation of 488 nm and emission of 525 nm. The level of DCF (i.e. fluorescence) was correlated to the level of ROS in the cell lysate samples, collected from the experiments. To measure whether MWCNTs themselves interfere the oxidization of H_2_DCF to DCF, the cell lysate samples with the newly added MWCNTs at 5, 10, and 20μg/ml were tested using the same procedure.

### GSH measurement

GSH level was measured from the cell lysate of A549 cells using GSH-GloTM Glutathione Assay (Promega, WI) to characterize the intracellular level of oxidative stress. The unit of GSH measurement was micro-molar (μM).

### Total protein measurement

Cells grown on each well (9.6 cm^2^) of BioFlex plates were lysed using 250 μl of RIPA buffer with protease inhibitors (Thermo Scientific, IL). Total amount of protein from cell lysate of each sample was measured using the BCA total protein assay (Pierce, IL) to evaluate cell proliferation. The unit of total protein was micrograms/milliliter (μg/ml).

### Statistical analysis

All data from IL-8, ROS and GSH measurement were normalized with total amount of protein measured from cell lysate, collected from respective samples. Statistical analyses were carried out using two-way analyses of variance (ANOVA) followed by Dunnett’s multiple comparison tests to determine where significance exists (p < 0.05). All graphs were prepared by plotting mean data (sample size, n = 3) with corresponding standard error of mean.

## Results

### Effect of MWCNT exposure on A549 cell growth

**Figure 2:**
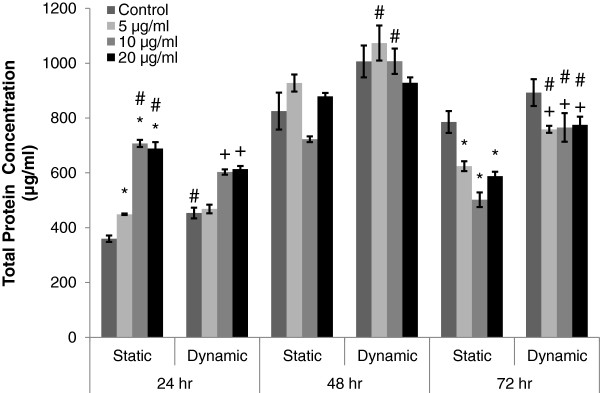
**Cell proliferation following exposure of different concentrations of MWCNTs under static and dynamic cell growth conditions.** Total protein concentration was measured in each cell lysate of A549 cell culture following exposure of MWCNTs at different concentrations (0, 5, 10, and 20 μg/ml) under both cell growth conditions. Time 0 h refers to the starting point of cyclic equibiaxial deformation. #Significantly different from the other cell growth condition at the same MWCNT concentration and exposure duration (p<0.05). *Significantly different from the control for static cell growth condition (static condition at 0 μg/ml of MWCNT) under the same exposure duration (p<0.05). +Significantly different from the control for dynamic cell growth condition (dynamic condition at 0 μg/ml of MWCNT) under the same exposure duration (p<0.05).

Total protein concentration was measured at every 24 hour interval following exposure of MWCNTs at different concentrations (0, 5, 10, and 20 μg/ml) to A549 cell monolayer under static or dynamic cell growth conditions (Figure 2). A549 cell proliferation increased as MWCNT concentration increased following 24 hour exposure of MWCNTs under both cell growth conditions (Figure 2, *p<0.05 and +p<0.05). Following exposure of lower concentration (5 μg/ml) of MWCNTs in dynamic cell growth condition, A549 cell proliferation was not higher than the control, and not different from that in static cell growth condition. However, following exposure of higher concentrations (10 and 20 μg/ml) of MWCNTs, A549 cell proliferation under static cell growth condition was significantly higher than that under dynamic cell growth conditions (Figure 2, #p<0.05). Following 48 hour exposure, MWCNT exposure did not induce any significant change in A549 cell proliferation as MWCNT concentrations increased under both cell growth conditions (Figure 2). However, cell proliferation following exposure of MWCNTs at 5 and 10 μg/ml in dynamitic cell growth condition was significantly higher than that in static cell growth condition (Figure 2, #p<0.05). Following 72 hour exposure of MWCNTs at all concentrations, A549 cell proliferation significantly decreased in both cell growth conditions (Figure 2, *p<0.05 and +<0.05). However, the A549 cell proliferation in dynamic cell growth condition remained significantly higher than that in the static cell growth condition following 72 hour exposure of MWCNTs at all concentrations (Figure 2, #p<0.05).

### Effect of MWCNT exposure on cellular inflammation

**Figure 3:**
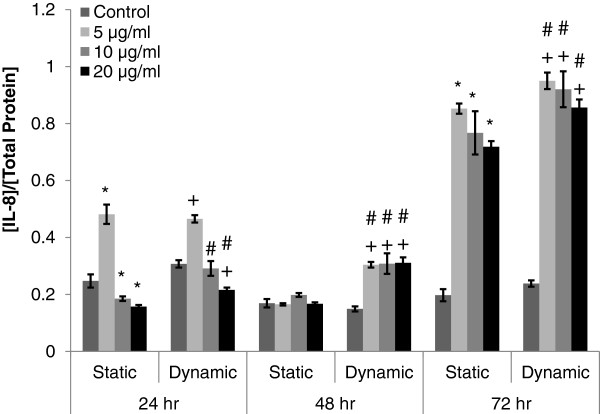
**Cellular inflammation in A549 cells following exposure of different concentrations of MWCNTs under static and dynamic cell growth conditions.** IL-8 was measured from the media supernatant of A549 cell culture following exposure of MWCNTs at different concentrations (0, 5, 10, and 20 μg/ml) under both cell growth conditions. Each data point from IL-8 measurement (pg/ml) was normalized by total protein concentration (μg/ml) at each time point. Time 0 h refers to the starting point of cyclic equibiaxial deformation. #Significantly different from the other cell growth condition at the same MWCNT concentration and exposure duration (p<0.05). *Significantly different from the control for static cell growth condition (static condition at 0 μg/ml of MWCNT) under the same exposure duration (p<0.05). +Significantly different from the control for dynamic cell growth condition (dynamic condition at 0 μg/ml of MWCNT) under the same exposure duration (p<0.05).

IL-8 level was measured at 24 hour interval from the media supernatant of A549 cultures following exposure of MWCNTs under either static or dynamic cell growth condition, to characterize the level of MWCNT-induced inflammation (Figure 3). Following 24 hour exposure of MWCNTs to A549 cells, IL-8 levels increased at 5 μg/ml of MWCNTs and decreased at 10 and 20 μg/ml of MWCNTs when compared to the control group under both cell growth conditions (Figure 3, *p<0.05, and +p<0.05), except at 10 μg/ml under dynamic cell growth condition. A549 cells grown in dynamic cell growth condition induced significantly higher level of IL-8 than that in static cell growth condition following 24 hour exposure of MWCNTs at 10, and 20 μg/ml (Figure 3, # p<0.05). Following 48 hour exposure of MWCNTs, IL-8 levels in static cell growth condition, were not significantly changed (Figure 3) whereas the IL-8 levels in dynamic cell growth condition significantly increased at all MWCNT concentrations when compared with controls in dynamic (Figure 3, +p<0.05) and static (Figure 3, #p<0.05) cell growth conditions. After 72 hour exposure of MWCNTs at all concentrations, IL-8 levels significantly increased in both cell growth conditions as compared to the controls (Figure 3, *p<0.05, and +p<0.05). IL-8 level in dynamic cell growth condition was significantly higher than that in static cell growth condition following 72 hour exposure of MWCNTs (Figure 3, #p<0.05).

### Characterization of oxidative stress by H_2_DCF measurement

**Figure 4:**
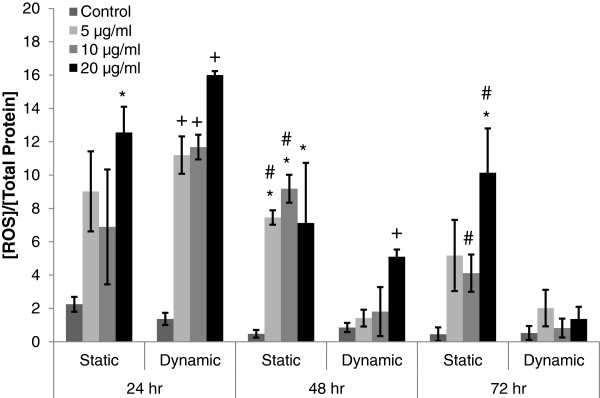
**ROS production in A549 cells following exposure of different concentrations of MWCNTs under static and dynamic cell growth conditions.** ROS was measured from the cell lysate of A549 cultures following exposure to MWCNTs at different concentrations (0, 5, 10, and 20 μg/ml) under both cell growth conditions. Each data point from ROS measurement (RFU) was normalized by total protein concentration (μg/ml) at each time point. Time 0 h refers to the starting point of cyclic equibiaxial deformation. #Significantly different from the other cell growth condition at the same MWCNT concentration and exposure duration (p<0.05). *Significantly different from the control for static cell growth condition (static condition at 0 μg/ml of MWCNT) under the same exposure duration (p<0.05). +Significantly different from the control for dynamic cell growth condition (dynamic condition at 0 μg/ml of MWCNT) under the same exposure duration (p<0.05).

ROS level was measured at 24 hour interval from the cell lysate of A549 cells following exposure of different concentrations of MWCNTs under either static or dynamic cell growth condition, to characterize the cellular level of oxidative stress (Figure 4). Following 24 hour exposure of MWCNTs to A549 cells, ROS levels in dynamic cell growth condition significantly increased as compared to the control (Figure 4, +p <0.05), while ROS levels in static cell growth condition did not increase significantly, except those at 20 μg/ml of MWCNTs, as compared to the control (Figure 4, *p<0.05). No significant differences in ROS level were observed between dynamic and static cell growth conditions following 24 hour exposure of MWCNTs. Following 48 hour exposure of MWCNTs to A549 cells, ROS levels in static growth condition significantly increased as compared to the control (Figure 4, *p<0.05), while ROS level in dynamic cell growth condition did not increase significantly, except that at 20 μg/ml of MWCNTs (Figure 4, #p<0.05). Under dynamic cell growth condition, Only 20 μg/ml of MWCNTs induced significantly higher level of ROS than the control after 48 hour exposure (Figure 4, +p<0.05). Following 72 hour exposure of MWCNTs to A549 cells in static cell growth condition, only 20 μg/ml of MWCNTs induced higher level of ROS than the control (Figure 4, *p<0.05). Moreover, ROS levels in static cell growth condition were significantly higher than those in dynamic cell growth condition, following 72 hour exposure of MWCNTs at 10 and 20 μg/ml (Figure 4, #p<0.05).

### Characterization of oxidative stress by GSH measurement

**Figure 5:**
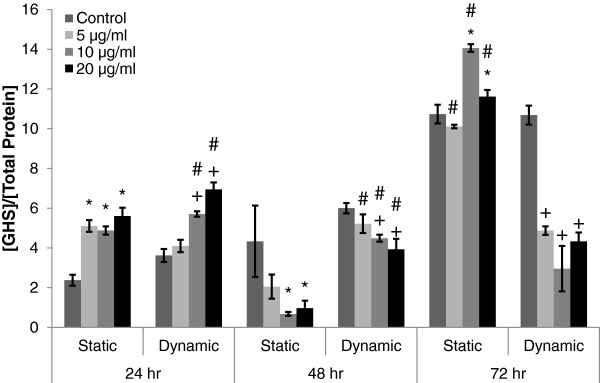
**GSH production in A549 cells following exposure of different concentrations of MWCNTs under static and dynamic cell growth conditions.** GSH was measured from the cell lysate of A549 cell cultures following exposure of MWCNTs at different concentrations (0, 5, 10, and 20 μg/ml) under both cell growth conditions. Each data point from GSH measurement (μM) was normalized by total protein concentration (μg/ml) at each time point. Time 0 h refers to the starting point of cyclic equibiaxial deformation. #Significantly different from the other cell growth condition at the same MWCNT concentration and exposure duration (p<0.05). *Significantly different from the control for static cell growth condition (static condition at 0 μg/ml of MWCNT) under the same exposure duration (p<0.05). +Significantly different from the control for dynamic cell growth condition (dynamic condition at 0 μg/ml of MWCNT) under the same exposure duration (p<0.05).

GSH level was measured from the cell lysate of A549 cells following exposure of different concentrations of MWCNTs in static or dynamic cell growth condition, to characterize the intracellular level of oxidative stress (Figure 5). Following 24 hour exposure of MWCNTs, GSH levels increased in both cell growth conditions as compared to each control, except those at 5 μg/ml of MWCNTs in dynamic cell growth condition (Figure 5, *p<0.05 and +p<0.05). 10 and 20 μg/ml of MWCNTs in dynamic cell growth condition induced significantly higher level of GSH than those in static cell growth condition after 24 hour exposure of MWCNTs (Figure 5, #p<0.05). Following 48 hour exposure of MWCNTs, 10 and 20 μg/ml of MWCNTs in both static and dynamic cell growth conditions induced significantly lower level of GSH than the control of each cell growth condition (Figure 4, *p<0.05 and *p<0.05). However, GSH level in dynamic cell growth condition was significantly higher than those in static cell growth condition (Figure 5, #p<0.05). Following 72 hour exposure of MWCNTs, GSH levels in dynamic cell growth condition significantly decreased as compared to the control (Figure 5, +p<0.05), while GSH levels in static cell growth condition significantly increased as compared to the control, except those at 5 μg/ml of MWCNTs (Figure 5, *p<0.05). Overall levels of GSH in static cell growth condition were significantly higher than those in dynamic cell growth condition following 72 hour exposure (Figure 5, #p<0.05).

## Discussion

In this study, we provided one of the alternative methods for the evaluation of MWCNT-induced effects on cellular responses such as cell proliferation, inflammatory responses, and oxidative stress. A dynamic cell growth environment was established to mimic the dynamic changes in the amount of circumferential and longitudinal expansion and contraction occurred during normal breathing movement in the lung. Dynamic cell growth environment may provide a realistic condition for facilitating interaction between nanomaterials and cells, nanomaterials uptake, and hence their effects on cells similar to *in vivo*[19,20,24,25]. We used Flexcell Tension Plus System to implement 5% cyclic equibiaxial elongation, which is equivalent to 45% of total lung capacity and the amount of stretching experienced during normal breathing condition [21]. Moreover, the equibiaxial elongation frequency was set as 0.2 Hz, which is corresponding to the normal human breathing rate. Under the highest MWCNT exposure concentration (20 μg/ml) in this study, the cell growth medium contained 0.668 μg/ml of carbon black, 0.094 μg/ml of chlorine, 0.048 μg/ml of iron, and 0.188 μg/ml of nickel. Such levels of iron and nickel impurities did not induce any toxicological effects on cells [26,27]. Our study indicated that MWCNT exposure induced significant changes in cell proliferation, cellular inflammation, and oxidative stress in A549 cell cultures in both cell growth conditions (Figures 2, 3, 4, and 5). In both cell growth conditions, A549 cell proliferation significantly increased following 24 hour exposure of MWCNTs at all concentrations as compared to the respective controls, except that at 5 μg/ml of MWCNTs in dynamic cell growth condition (Figure 2). Increased cell proliferation and decreased IL-8 level (Figure 3) might have been related to the increased level of in GSH during 24 hour exposure of MWCNTs (Figure 5). Kang et al. and Horton et al. have demonstrated the increasing GSH levels with A549 cell proliferation, which could explain on the increased cell proliferation during 24 hour exposure of MWCNTs [28-31]. Similarly, the intracellular GSH inhibit IL-8 expression by inhibiting nuclear factor-kappaB (NF-κB) activation [32,33]. The possibility of interaction between IL-8 and MWCNTs should not be ruled out to explain the results during 24 hour exposure of MWCNTs [34,35]. During 24 hour exposure, the levels of ROS significantly increased (Figure 4, +p<0.05) in dynamic cell growth condition. Increased level of ROS reduced cell viability and increased NF-κB mediated IL-8 up-regulation [36-38]. Following 48 hour exposure of MWCNTs, cell proliferation was not significantly changed as MWCNT concentration increased in each cell growth condition (Figure 2). However, A549 cell proliferation in dynamic cell growth condition was significantly higher than that in static cell growth condition (Figure 2, #p<0.05). Similar trends were observed in the IL-8 (Figure 3, #p<0.05) and GSH levels (Figure 5, #p<0.05), which were significantly higher in dynamic cell growth condition than static cell growth condition. In both cell growth conditions, GSH levels decreased as MWCNT concentration increased and were significantly lower at higher MWCNT concentrations (10, and 20 μg/ml) (Figure 5, *p<0.05, and +p<0.05). While GSH level was decreasing, ROS level was increasing in static cell growth condition (Figure 4, *p<0.05), which indicated the increase in oxidative stress. However, ROS level in dynamic cell growth condition remained significantly lower at all concentrations of MWCNTs (Figure 4, #p<0.05), except that at 20 μg/ml of MWCNTs at which ROS level was significantly higher than the control, but not significantly different from that at same concentration of MWCNTs in static cell growth condition. Increased level of oxidative stress might have down-regulated cell proliferation in static cell growth condition. After 72 hour exposure of MWCNTs, A549 cell proliferation was significantly lower in both cell growth conditions than the respective controls (Figure 2, *p<0.05, and +p<0.05). A549 cell proliferation in dynamic cell growth condition was significantly higher than that in static cell growth condition (Figure 2, #p<0.05). A549 cell proliferation in static cell growth condition might have been down-regulated by the increased levels of ROS (Figure 4, *p<0.05) and IL-8 (Figure 3, *p<0.05). Similarly, the decreased cell proliferation in dynamic cell growth condition might have been due to the reduced levels GSH (Figure 5, +p<0.05) and increased levels of IL-8 (Figure 3, +p<0.05). During the same exposure time, the levels of IL-8 significantly increased in both cell growth conditions, which might have resulted from the prolonged MWCNT exposure (Figure 3, *p<0.05, and +p<0.05). In dynamic cell growth condition, the level of IL-8 was significantly higher than that in the static cell growth condition (Figure 3, #p<0.05). A549 cell proliferation generally decreased as MWCNT concentrations increased during longer exposure time (48 and 72 hours) in both cell growth conditions. During the same exposure time (48 and 72 hours), A549 cell proliferation in dynamic condition was significantly higher. IL-8 level increased as MWCNT concentrations increased during longer exposure time (72 hours) in both cell growth conditions (Figure 3). Increased level of IL-8 can be related to neutrophil migration and mucin production as precursory events to remove MWCNTs from sites of inflammation in the lung [20,39,40]. Dynamic cell growth condition facilitated a significant increase in IL-8 level following 48 hour exposure (Figure 3, +p<0.05, and #p<0.05), which was similar to the results from animal studies indicating the recruitment of neutrophil in bronchoalveolar lavage (BAL) fluid within 48 hour of MWCNT exposure [6,10,41]. ROS level decreased over the exposure duration in dynamic cell growth condition, whereas it remained increasing in static cell growth condition. After initial significant increase in ROS levels following 24 hour exposure of MWCNTs, ROS levels were reduced to the lower level over the exposure duration in dynamic cell growth condition (Figure 4), which was similar to the results from animal study performed by Han et al. [6]. Similarly, GSH levels decreased over the exposure duration in dynamic cell growth condition (Figure 5). However, the level of effects was not just due to dose–response as exposure time increase. Especially, following 72 hour exposure, GSH levels in dynamic cell growth condition dramatically decreased as MWCNT concentration increased, while GSH levels in static cell growth condition still increased as MWCNT concentration increased. For the longer exposure at higher concentration of MWCNTs induced more distinct difference in GSH levels between static and dynamic cell growth conditions. Our results strongly demonstrated the distinct differences in MWCNT-induced effects on cell proliferation, IL-8, ROS, and GSH between static and dynamic cell growth conditions. Interestingly, ROS and IL-8 levels in dynamic condition were found to be similar to the results from animal studies.

## Conclusions

The dynamic cell growth system together with static cell growth system yielded several important findings: (1) All MWCNT exposure concentrations used in this study affected A549 cell proliferation. Cell proliferation in dynamic cell growth condition was higher than static cell growth condition during 48 and 72 hour exposure (Figure 2). (2) IL-8 levels were significantly higher in dynamic cell growth condition than static cell growth condition, except those at 5μg/ml of MWCNTs after 24 hour exposure (Figure 3). (3) ROS and GSH levels were relatively lower in dynamic cell growth conditions than those in static cell growth condition during longer exposure time (Figures 4 and 5). The dynamic cell growth system used in this study provided important changes in cellular responses that were not found in the static cell growth system and similar to the results from animal studies. The dynamic cell growth system can be considered as a viable alternative to *in vivo* test system in combination with existing *in vitro* static cell growth systems to evaluate the effect of MWCNTs on cellular responses in the respiratory system.

## Competing interests

The authors declare that they have no competing interest.

## Authors’ contributions

HP performed experimental design and data analysis for the research presented in the paper. SK have funded, designed, and significantly contributed to the writing of the research presented in the paper. All authors have read and approved the final manuscript.
